# Editorial: Entomopathogenic Fungi for the Control of Arthropod Pests

**DOI:** 10.3389/fphys.2022.885131

**Published:** 2022-03-25

**Authors:** Isabele C. Angelo, Jose Luiz Ramirez, Patricia S. Golo, Éverton K. K. Fernandes, Nicolás Pedrini, Vânia R. E. P. Bittencourt

**Affiliations:** ^1^ Departamento de Epidemiologia e Saúde Pública, Instituto de Veterinária, Universidade Federal Rural Do Rio de Janeiro, Seropédica, Brazil; ^2^ Crop Bioprotection Research Unit, National Center for Agricultural Utilization Research, Agricultural Research Service, United States Department of Agriculture, Peoria, IL, United States; ^3^ Departamento de Parasitologia Animal, Instituto de Veterinária, Universidade Federal Rural do Rio de Janeiro, Seropédica, Brazil; ^4^ Departamento de Biociências e Tecnologia, Instituto de Patologia Tropical e Saúde Pública, Universidade Federal de Goiás, Goiânia, Brasil; ^5^ Instituto de Investigaciones Bioquímicas de La Plata (INIBIOLP) - Universidad Nacional de La Plata, La Plata, Argentina

**Keywords:** biological control, Metarhizium anisopliae, Beauveria bassiana, environmentally-friendly agents, biopesticide

Biological control has stood out, particularly in recent years, as a sustainable and environmental-friendly method to control arthropod pests that cause significant economic losses in agriculture, and negatively impact animal and human health. This Research Topic encompasses the contribution of eleven original research articles/reviews that demonstrate the use of entomopathogenic fungi (EPF), especially *Metarhizium anisopliae* and *Beauveria bassiana,* to control arthropod pests with relevance in agriculture (*Athalia rosae*, *Solenopsis invicta* and *Oedalus asiaticus*), animal health (*Stomoxys calcitrans, Rhipicephalus microplus* and *Haemaphysalis longicornis*), and public health (*Anopheles* sp. and *Aedes aegypti* mosquitoes). In addition, this collection includes two original research articles that expand our knowledge on the molecular mechanisms underpinning blastospores’ and microsclerotia development and provide insights into microsclerotia tolerance to ultraviolet B (UV-B) radiation and heat.

The virulence of EPF depends on the infectivity of their conidia or blastospores, propagules with distinct morphological and physiological characteristics (Mascarin et al., 2019) that in turn present advantages and disadvantages to their use (de Paula et al., 2021). In this Research Topic, Paixão et al. describe two other *Metarhizium robertsii* propagules (microsclerotia and mycelial pellets) that are morphologically similar but differ in biomass production and tolerance to UV-B radiation and heat. This study also characterized the ultrastructure and gene expression pattern involved in microsclerotial differentiation.

Blastospores of *B. bassiana* are promising propagules for pest control. Their production is conducted via liquid fermentation with high glucose concentration and high aeration. However, the mechanisms behind the development of these propagules are not completely understood. Mascarin et al. conducted an RNAseq-based transcriptomic study of *B. bassiana* blastospores and showed that a higher proportion of genes were downregulated when the fungus was grown under high glucose than under low glucose concentrations. However, other genes related to the antioxidant response, calcium transport, conidiation, and osmosensory signaling, were highly upregulated in high glucose concentrations. These molecular findings provide new knowledge on blastospore development and may help facilitate the industrial production of *B. bassiana* blastospores for a wide range of pest control applications.

The mechanism of EPF infection of a host is complex and while some advancements have been made, there is still much more that needs to be uncovered to improve biological control efforts. On this end, recent findings indicate that the Msn2 transcription factor regulates the acaricidal virulence in the fungal pathogen *B. bassiana*. Loss of the Msn2 transcription factor in *B. bassiana* mutants caused reduced production of protease, which may have contributed to the inability of the mutant strain to breach the cuticle of the tick *R. microplus* (Muniz et al.). Two other articles reported the use of EPF against ticks (Alonso-Dias and Fernández-Salas and Lee et al.). The first is a review on the acaricidal effect of fungi to ticks with relevance for Mexico’s cattle livestock, particularly *R. microplus*, *Rhipicephalus annulatus*, and *Amblyomma mixtum*. These authors highlighted that epidemiological and environmental aspects are important for the EPF acaricidal efficacy. In turn, Lee et al. analyzed the molecular interactions at the transcriptional level between the longhorned tick *H. longicornis* and *M. anisopliae*. These authors reported that both fungus and tick genes were mostly up-regulated at the early stages of infection. This suggests that while the fungus starts the infection process, the tick is actively mounting a defense response.

The stable fly, *Stomoxys calcitrans,* is another arthropod, along with ticks, that significantly affects the livestock industry. A study by Baleba et al. assessed the infection of the stable fly by *M. anisopliae* and provided detailed information on how fungal infection affects the feeding, fecundity, and fertility of this harmful fly. Among eleven fungal strains screened, one of them was identified as the most virulent, with potential to be developed as a biopesticide agent against this fly*.*

Entomopathogenic fungi are also regarded as potential biocontrol agents against major arthropod vectors of human pathogens. Two articles in this Research Topic describe new efforts on this front, providing a potential new delivery method and identifying new fungal species with biocontrol potential. Here, Reyes-Villanueva et al. described the contact rate of wild *A. aegypti* females with fungus-infected males, providing support to a mosquito control strategy via the release of fungus-infected males. Meanwhile, Accoti et al. conducted an environmental screen and described five fungi with pathogenicity against two major mosquito vectors.

Smaller in size, in comparison to the other arthropod pests described above, the fire ant, *S. invicta*, is a major urban and agricultural pest. A study by Wei et al. examined the temporal gene expression profiles of chemosensory and odorant binding proteins (CSPs and OBPs, respectively) in response to infection by *B. bassiana*. Their study describes the dynamic gene expression of these two gene families, providing insights into the mechanisms that might mediate detection of microbial pathogens, and would trigger grooming and nest sanitation. In a study that evaluated the use of EPF against another hymenopteran, the sawfly *A. rosae*, Zanchi et al. demonstrated that this insect pest is resistant to *B. bassiana* infection, with low incidence of mycelial growth and sporulation from *A. rosae* cadavers. Furthermore, their results revealed that clerodanoids, compounds with antimicrobial activity adsorbed by *A. rosae* adults from host plants, are unlikely to be responsible for their resistance against this EPF.

Entomopathogenic fungi are often tested in association with plant-derived metabolites in an effort to increase their lethality against insect pests. In this regard, plant-derived oils can provide added protection to the propagule and maintain EPF virulence. In such study, Li et al. evaluated the synergistic control effect of *artemisia sieversiana* crude extracts with *M. anisopliae* on *O. asiaticus*, a major pest in northern China. By using different doses of *M. anisopliae* and crude extracts of *A. sieversiana,* singly and in combination, and by analyzing four insect enzymes, the authors demonstrated that *A. sieversiana* effectively increases *M. anisopliae* virulence.

The review articles summarize the current state of the art and the original articles included in this Research Topic provide much-needed new knowledge in this area of research and will improve the use of EPF as biocontrol agents against important arthropod pests. We thank all authors for their contribution and participation in this Research Topic. We are also grateful to all reviewers and editors, whose participation during the publication process made the development of this Research Topic possible.

## References

[B1] de Paula A. R. Silva L. E. I. Ribeiro A. da Silva G. A. Silva C. P. Butt T. M. (2021). Metarhizium Anisopliae Blastospores Are Highly Virulent to Adult *Aedes aegypti*, an Important Arbovirus Vector. Parasites Vectors 14, 555. 10.1186/s13071-021-05055-z 34711272 PMC8555014

[B2] Mascarin G. M. Lopes R. B. Delalibera Í. Fernandes É. K. K. Luz C. Faria M. (2019). Current Status and Perspectives of Fungal Entomopathogens Used for Microbial Control of Arthropod Pests in Brazil. J. Invertebr. Pathol. 165, 46–53. 10.1016/j.jip.2018.01.001 29339191

